# Synthetic Strategies toward High Entropy Materials: Atoms-to-Lattices for Maximum Disorder

**DOI:** 10.1021/acs.cgd.3c00712

**Published:** 2023-08-31

**Authors:** Mark A. Buckingham, Jonathan M. Skelton, David J. Lewis

**Affiliations:** † Department of Materials, The University of Manchester, Oxford Road, Manchester, M13 9PL, United Kingdom; ‡ Department of Chemistry, The University of Manchester, Oxford Road, Manchester, M13 9PL, United Kingdom

## Abstract

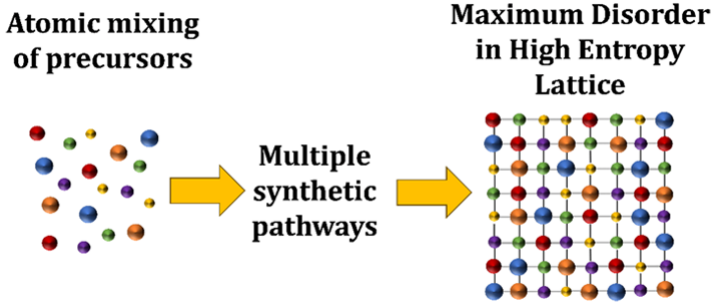

High-entropy materials are a nascent class of materials that exploit a high configurational entropy to stabilize multiple elements in a single crystal lattice and to yield unique physical properties for applications in energy storage, catalysis, and thermoelectric energy conversion. Initially, the synthesis of these materials was conducted by approaches requiring high temperatures and long synthetic time scales. However, successful homogeneous mixing of elements at the atomic level within the lattice remains challenging, especially for the synthesis of nanomaterials. The use of atom-up synthetic approaches to build crystal lattices atom by atom, rather than the top-down alteration of extant crystalline lattices, could lead to faster, lower-temperature, and more sustainable approaches to obtaining high entropy materials. In this Perspective, we discuss some of these state-of-the-art atom-up synthetic approaches to high entropy materials and contrast them with more traditional approaches.

## Abstract 2

In this Perspective Lewis and co-workers discuss some of the state-of-the-art atom-up synthetic approaches toward high entropy inorganic materials, comparing and contrasting them with more traditional methods.

## Defining High-Entropy Materials

High-entropy (HE) materials are those which contain multiple elements in a single crystal lattice (e.g., in multicomponent alloys) or sublattice (e.g., in metal oxides or metal chalcogenides).^1−5^ The presence of multiple elements gives rise to a multitude of possible atomic arrangements, yielding disorder and a high configurational entropy *S*_conf_ that can be calculated as
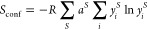
1where *R* is the gas constant (8.314 J K^–1^ mol^–1^), *a*^*S*^ is the fraction of each sublattice *S* in the overall composition, and *y*_*i*_^*S*^ is the mole fraction of each constituent element *i* occupying *S*.^6^ In a single lattice system such as an alloy, with an equimolar concentration of elements, this equation can be simplified to
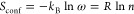
2where *n* is the number of elements present, *k*_B_ is the Boltzmann constant, and ω is the number of ways of mixing.^7^

**Figure 1:**
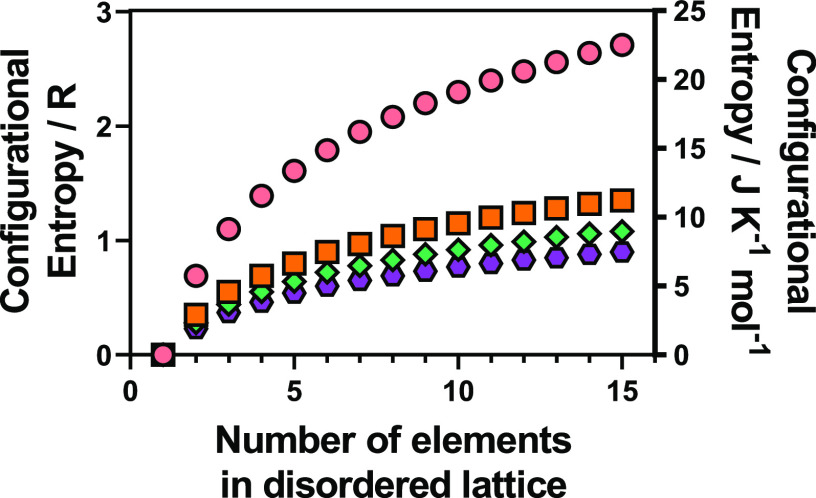
Calculated configurational entropy as a function of the number of elements present in equimolar concentrations for an alloy M in a single disordered sublattice (pink circles) and a range of common metal–chalcogenide materials with a single chalcogenide sublattice (i.e., X = O, S, Se, or Te) and a disordered metal sublattice (M) with composition MX (orange squares), M_2_X_3_ (blue diamonds), and MX_2_ (purple hexagons). Figure reproduced with permission from ref (8) under a CC BY 3.0 license (https://creativecommons.org/licenses/by/3.0/).

In the context of HE materials, *high entropy* has historically been regarded as *S*_conf_ > 1.5 *R*. For an alloy with a single disordered sublattice, this value can be obtained with five elements present in equimolar ratios (Figure 1).^8^ However, it is not possible to reach this value with only a singly disordered, multilattice system such as a metal oxide or chalcogenide (Figure 1). We previously proposed that *high entropy* can be reached when 6 or more elements are present (at least 5 in a singly disordered sublattice, i.e., hexernary materials or higher), with each element prevalent with 5 mol % abundance or higher.^8^ It is also possible to achieve *S*_conf_ > 1.5 *R* in systems containing multiple disordered lattices, such as in the HE metal–chalcogenide (PbSnSb)(SSeTe).^9^ We note that this definition excludes doped materials, which are often labeled as “high entropy” in the literature.

The first reports of HE materials were a FeCrMnNiCo alloy by Cantor et al.^10^ and a CuCoNiCrAl_*x*_Fe HE alloy by Yeh et al., both in 2004.^11^ In the two decades since, the library of HE materials has been expanded beyond alloys and now includes oxides,^12,13^ chalcogenides,^8^ carbides,^14^ flourites,^15^ silicides,^16^ borides,^17^ nitrides,^18^ phosphides/phosphates,^19−21^ perovskites,^22,23^ and spinels,^24,25^ among others.^1^ Moreover, nanoscale HE materials including 0D nanoparticles,^26−30^ 1D nanowires,^31−33^ and 2D nanosheets^34−37^ have been developed in addition to bulk materials.

The consequences of being able to form stable, multielement compounds was summarized in a talk by Cantor to the University of Udine in 2021.^38^ Cantor considered multicomponent alloys containing *C* different chemical elements, each with mole fraction *x*, and defined unique materials as those differing by percentage increments in *x* such that the number of possible compositions, *n*, is given by
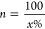
3

From this definition, the total number of chemically distinct materials, *N*, is then:
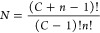
4Using this definition, if a conservative estimate that there are around 40 usable elements in the periodic table, and *x* = 1%, then the number of distinct chemical compounds in the high-entropy space could be as large as *N* = 10^30^. (As of 2023, many of the 118 known elements can be ruled out straightforwardly on the basis of inertia, transience, toxicity, radioactivity, etc.). To put this into perspective, the number of known stable materials discovered to date is around *N* = 10^12^. When the almost limitless stoichiometric space is combined with the diversity of material structures and length scales, the possibilities for producing distinct compounds with properties tailored to specific applications are both exciting and potentially boundless.

## Advantages of High-Entropy Materials

**Figure 2:**
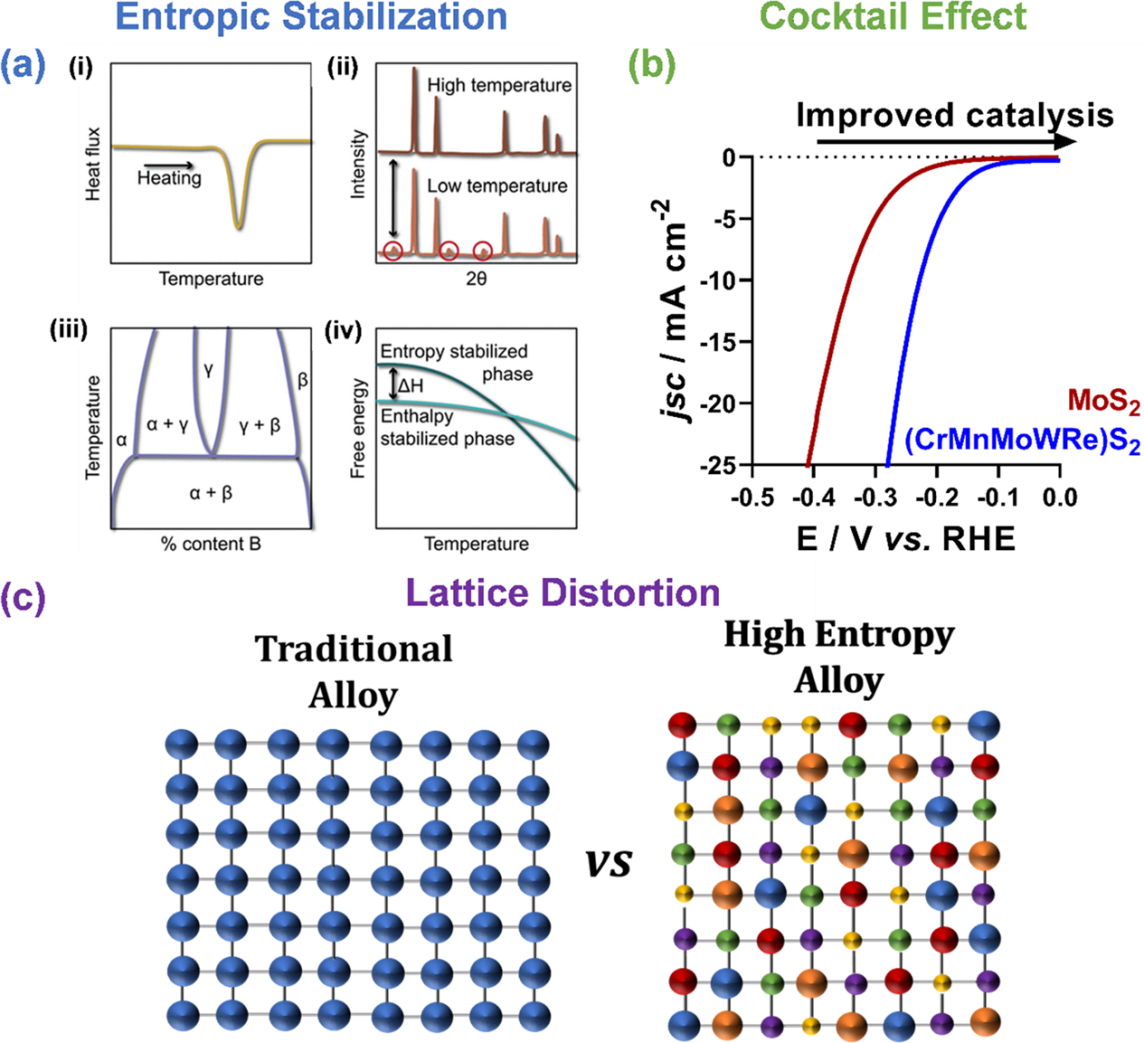
Example properties of high-entropy materials. (a) Entropic stabilization can be evidenced from multiple approaches including: (i) calorimetric verification of an endothermic heat of formation; (ii) demonstrating the reversibility of phase transformations through the appearance and disappearance of impurity peaks in X-ray diffraction patterns with heat treatment; (iii) formation of a cation-disordered phase in the center of the phase diagram in a structure that is distinct from any of the constituent phases; and (iv) calculating the enthalpy of formation Δ*H* of two competing entropy-stabilized and enthalpy-stabilized phases using DFT and demonstrating that the configurational entropy will dominate the free energy at some temperature below melting. Reprinted from ref (53). (b) Electrocatalytic performance of MoS_2_ and a high-entropy MoS_2_-structured system based on linear sweep voltammograms (LSVs) within the hydrogen evolution reaction potential range. Adapted with permission from ref (34) under a CC BY 4.0 license (https://creativecommons.org/licenses/by/4.0/). (c) Visual representation of how the presence of multiple elements of various sizes induces lattice distortion in a HE alloy compared to a single element traditional alloy.

HE materials show a number of unique and favorable properties over traditional materials, including entropy stabilization, sluggish diffusion, significant lattice distortion, and the so-called *cocktail effect* (Figure 2),^7^ which often lead to superior performance in certain applications compared to traditional materials.

Entropy stabilization is exploited in HE materials to stabilize phase-pure materials over multiphase materials.^39^ Entropy stabilization of HE materials has been demonstrated to enhance the stability of materials to harsh conditions: for example, (PtNiMgCuZnCo)O_*x*_ has been shown to be thermally stable up to 900 °C and to be reusable for at least 40 h, with high conversion rates (>90%) for thermal CO oxidation catalysis, compared to medium entropy equivalent oxides.^39^ Entropic effects can also stabilize materials in rare or unexpected phases.^2,40,41^ We note here that the distinction between high-entropy and entropy-stabilized materials is significant; a material can be both high-entropy and multiphase when the constituent elements are present in multiple crystal systems or polymorphs, whereas an entropy-stabilized material is strictly high-entropy material stabilized in a single polymorph by high configurational entropy.^12,39^

Sluggish diffusion occurs through anomalously slow diffusion kinetics thought to result from fluctuations in the potential energy at the lattice sites in HE materials.^7,42,43^ It should be noted, however, that some reports have found that diffusion in HE materials is not unusually slow, as demonstrated by observations of precipitation in some HE alloys, including those subjected to very rapid cooling or quenched following high-temperature heat treatment.^44,45^ Where they have been observed, these effects appear to be restricted to HE alloys and have not been observed in other inorganic HE materials.

Lattice distortion in high-entropy materials is caused by variation in the size of the multiple elements present within the crystal lattice (Figure 2c). This leads to distortion and strain that influence properties including thermodynamic stability, microstructure, and deformation mechanisms.^46^ The lattice distortion can be singularly beneficial, with a good example being the HE metal chalcogenide (PbSnSb)(SSeTe). In this system, the presence of elements of different sizes in both the cationic and anionic sublattices was found to significantly lower the thermal conductivity compared to the constituent binary materials due to disrupted phonon transport arising from the lattice distortion and increased strain.^9^ The electronic properties, on the other hand, are largely preserved, making this an example of a “phonon glass-electron crystal” (PGEC) material and leading to improved thermoelectric efficiency.^9^

The *cocktail effect* has been used as a blanket term to describe emergent properties of HE materials that cannot be attributed to their constituent components. This effect is particularly prevalent in catalysis, where the random distribution of elements in the lattice yields innumerable potential catalytic sites. Catalytic activity in principle depends on both the individual site and the surrounding elements, and synergy between different elements can enhance the surface activity.^47^Table 1 gives an estimate of the number of unique sites as a function of the number of elements in a multicomponent alloy and the number of nearest-neighbor atoms considered to influence a site, again based on statistical arguments made by Cantor.^38^ The results are striking. Considering a six-element HE material, with unique sites defined by the first-nearest neighbor only, yields an estimate of 10^10^ unique sites for catalysis. When the second- and third-nearest neighbors are considered to contribute to the catalytic properties, which is commonly the case, this rises exponentially to 10^14^ and 10^33^, respectively. In this respect, the use of high-entropy materials for catalysis could be viewed as combinatorial chemistry at the atomic scale. Although it should be noted that not all HE materials will be good catalysts; this is dependent on a wide range of factors including present surface elements, crystallographic lattice, material stability, catalytic sites, crystallographic defect sites, crystal facets, energy band gap, and adsorption energy.^48−52^

**Table 1:** An Estimate of the Unique Sites for Catalysis in a Multicomponent High-Entropy Alloy as a Function of the Number of Elements and the Number of Nearest Neighbors Considered to Contribute to the Activity of a Given Site

	nearest neighbors considered		
no. of unique elements in HE material	first	second	third
3	10^6^	10^9^	10^20^
4	10^7^	10^11^	10^25^
5	10^9^	10^13^	10^30^
6	10^10^	10^14^	10^33^

For example, the 2D HE metal disulfide (CrMnMoWRe)S_2_ shows significantly enhanced electrocatalytic hydrogen evolution performance compared to the parent MoS_2_ (Figure 2b).^34^ A combination of experimental and computational analysis showed that while the electrochemically active surface area increased by around 2×, the catalytic activity increased by 8×. Density-functional theory (DFT) calculations determined that the presence of Mn and Cr significantly reduced the adsorption energy *E*_ads_ for H to the basal plane, activating the previously inactive surface for catalysis. It is important to note that Mn is difficult to dope into MoS_2_ in high quantities, as it tends to form 3D MnS and destroys the layer structure. However, doping high levels of both Mn and Cr was achievable in this report through the entropy stabilization effect.^54^ The DFT calculations further showed that combinations of elements improved the *E*_ads_, even when only two elemental substitutions of Mo for Mn and Cr at the MoS_2_ surface were considered, demonstrating the synergistic effect of multiple elements within a local area.^34^

## Traditional Solid-State Synthetic Routes to HE Materials

Myriad approaches have been used for the synthesis of HE alloys, oxides, and chalcogenides, and have already been reviewed elsewhere in more detail than will be covered here.^8,55−57^

The seminal work on HE materials by Cantor and Yeh both employed electrical heating of the constituent elements.^10,11^ This elemental annealing approach represents a traditional solid-state synthetic strategy and has subsequently been widely employed for the synthesis of more complex materials such as oxides (annealed in air),^12,58^ chalcogenides (annealed with the chalcogen in an inert environment),^59^ and even mixed-anion systems (e.g., halides and phosphorus trisulfides).^35^ However, elemental annealing is both time-consuming and energy intensive. Taking oxides as an example, (MgNiCoCuZn)O can be prepared by combining the individual metal oxides in a shaker mill and milling for at least 2 h, followed by an annealing step from 750 to 900 °C in 50 °C increments, with phase-pure material being obtained between 850 and 900 °C.^12^ (NiMgCuZnCo)O has also been prepared by milling five metal salts (such as chlorides, nitrates, etc.) for at least 2 h, followed by calcination in air at 800, 900, and 1000 °C for 4 h.^39^ For metal chalcogenides, (MoWVNbTa)S_2_ can be prepared by a HF etching step and annealing at 1000 °C for 24 h to prepare the reaction vessel, followed by ramp annealing of elements at 466 °C for 120 h.^60^ (CoFeNi)(SSe) can be prepared by annealing the individual elements at 1000 °C for 24 h, followed by another annealing step at 500 °C for a further 72 h.^61^

At a fundamental level, elemental annealing amounts to taking a prefabricated lattice (an alloy, oxide, etc.), such as a purchasable metal salt, oxide, or sulfide, deconstructing it, and reconstructing a new HE lattice. To deconstruct prefabricated lattices with no disorder (such as binary metal oxides) and reconstruct a new HE lattice necessitates high-energy processes and long time scales. This is potentially problematic for the synthesis of HE materials for two reasons. First, a homogeneous distribution of the elements in the disordered lattice (or sublattice) is required in order to maximize the configurational entropy and consequent benefits, but it is challenging to achieve this without homogeneous mixing of the elements prior to building the HE lattice. This difficulty is the cause of the localized elemental clustering often observed in these systems, even after high-temperature and long-time annealing. Second, the enthalpy (Δ*H*) and entropy (Δ*S*) of mixing are in competition during the synthesis to obtain an overall negative Gibbs free energy of mixing Δ*G* as in eq 5:
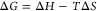
5

Another common solid-state synthetic approach is high-energy ball-milling, which has been used in the synthesis of high-entropy metal sulfides. For example, HE (FeCuMnNiCoTiCr)_*x*_S_*y*_ can be formed by ball milling FeS_2_, CuS, MnS, Ni_3_S_2_, CoS_2_, TiS_2_, Cr metal, and sulfur powder in the required metal-to-sulfur ratios for between 60 h (MS, M_2_S_3_, M_3_S_4_, and M_3_S_2_) and 110 h (MS_2_). However, despite the long reaction time, many of the synthesized samples were still found to be multiphasic, showing that obtaining a homogeneous distribution of elements in a single phase is still not possible with this approach.^62^

## HE Nanomaterial Synthesis Using Atomic Integration into Prefabricated Lattices

Solid-state synthetic strategies such as those discussed above are typically used to form bulk HE materials. However, some synthetic strategies for obtaining nanocrystalline HE materials involve a similar approach of starting from a prefabricated lattice but critically use atomic-scale approaches to introduce disorder to form HE lattices.

**Figure 3:**
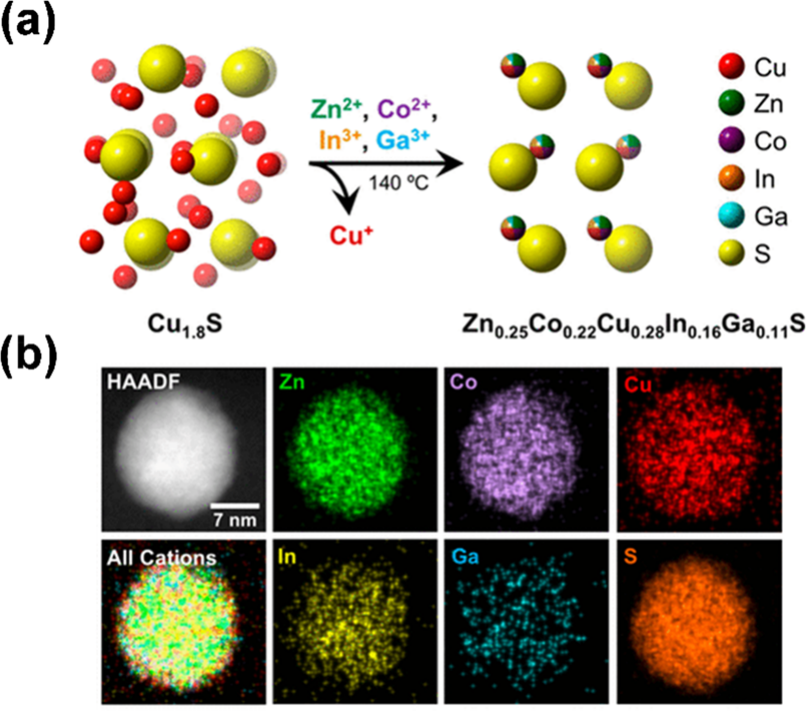
(a) Schematic showing the formation of high-entropy wurtzite-type metal sulfide (ZnCoCuInGa)S from simultaneous Zn^2+^, Co^2+^, In^3+^, and Ga^3+^ exchange with the Cu^+^ cations in roxbyite Cu_1.8_S. (b) Characterization of a single (ZnCoCuInGa)S nanoparticle and corresponding STEM-EDX element maps. Adapted from ref (26).

An interesting example of this strategy is the cation-exchange method. HE (ZnCoCuInGa)S nanoparticles can be prepared from prefabricated roxbyite Cu_1.8_S nanoparticles prepared by using solvothermal synthesis. Cation-exchange is then achieved via a sequence of solvothermal heating steps in the presence of further metal salts to exchange the Cu ions for Zn, Co, In, and Ga (Figure 3).^26^ The resulting HE nanoparticles were found to possess a hexagonal wurtzite-type structure, differing significantly from the triclinic starting material. There are, however, some considerations that need to be addressed using this method. To facilitate the exchange, a soft base such as TOP is used to coordinate the leached soft acid Cu^+^, which aids in integrating the harder acid divalent and trivalent Zn^2+^, Co^2+^, In^3+^, and Ga^3+^ ions into the nanocrystal. The authors also noted that the Cu^+^, Zn^2+^, Co^2+^, and In^3+^ have similar sizes in the tetrahedral coordination environment, although they propose that this does not play a key role in the cation-exchange mechanism.^26^

**Figure 4:**
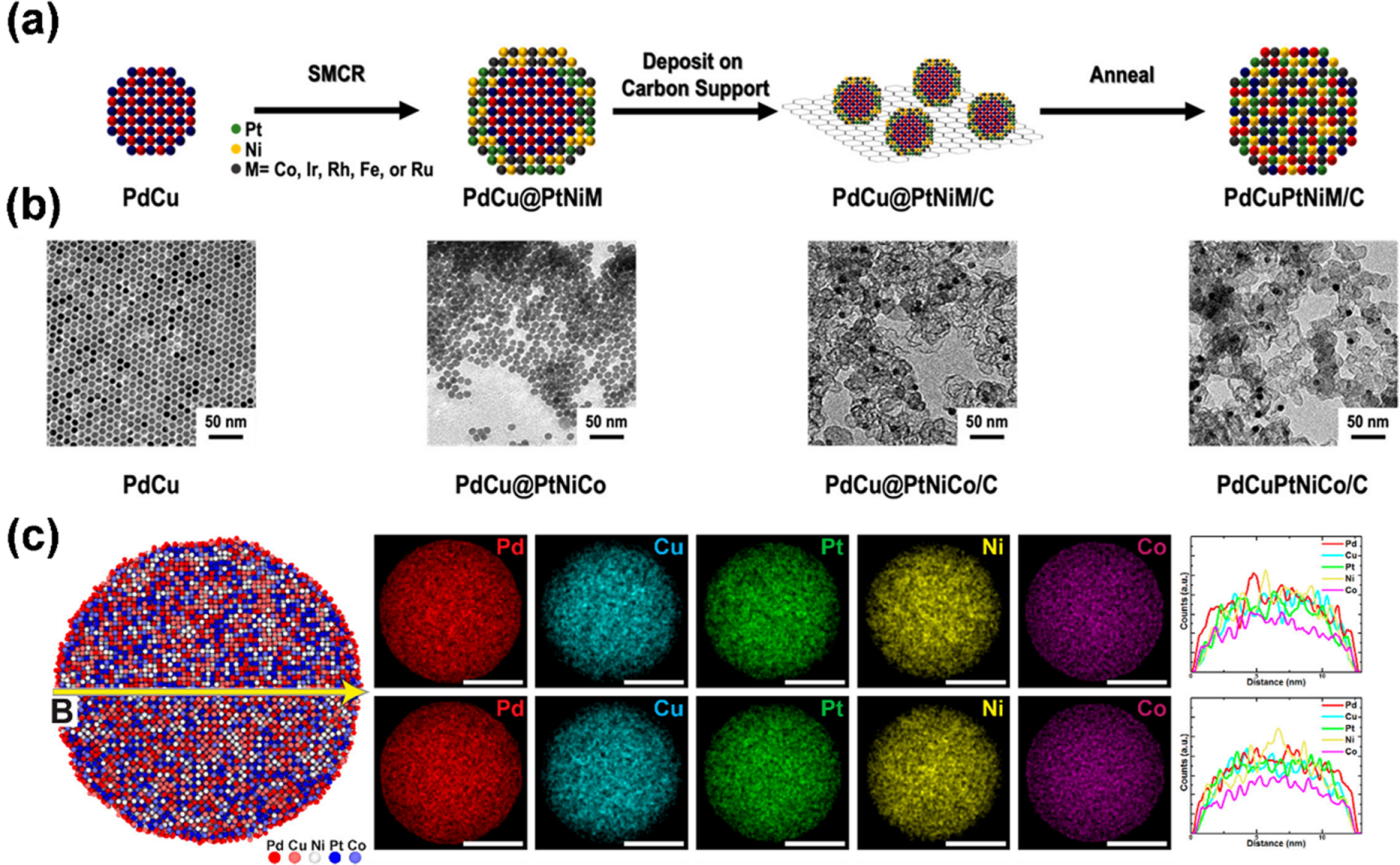
(a) Illustration of the three-step process used to obtain HEA NPs with (b) TEM images of the starting material, intermediates, and PdCuPtNiCo product. (c) Atomistic simulations of the phase stability of the quinary PdCuPtNiCo HEA NPs: cross-section of the structure (left column), simulated STEM-EDS elemental mapping (middle column), and line scans (right column). Adapted from ref (63).

Another interesting atomistic approach toward the synthesis of HE nanomaterials is through the prefabrication of core@shell nanoparticles, followed by an annealing step to homogenize the core@shell structure to a high-entropy nanoparticle (Figure 4).^63,64^ This method entails several synthesis, purification, and annealing steps. In one method, Pd and Cu salts were annealed at 235 °C for 30 min in an oleylamine/oleic acid solution with added TOP under Ar. The resulting PdCu seeds were then collected by centrifugation and added to an oleylamine/1-octadecene mixture containing salts of Pt, Co, and Ni and 1,2-dodecanediol and heated first to 110 °C for 30 min and then to 235 °C for a further 30 min. Quinary PdCu@PtNiCo NPs were then collected by centrifugation, deposited on a carbon support, sonicated for 1 h, and left to stir overnight. Finally, a fused silica tube containing the sample was purged with a H_2_/N_2_ (4 v/v %) mixture for 30 min and annealed at 600 °C for 10 h, yielding quinary PdCuPtNiCo HE NPs.^63^

While these represent interesting approaches toward the synthesis of HE nanoparticles, there are again considerations that need to be addressed. For the element-exchange method, the main limitation is the length scale of diffusion over the reaction time. With a typical diffusion length of *ca*. 15 nm in 25 min, it would require *ca*. 115 days for diffusion over 100 μm, making this approach impractical for producing bulk HE materials.^8^ The core@shell approach requires lower temperatures and is faster than elemental annealing, but it is still labor, time, and energy intensive and is only conducive to the synthesis of nanoparticulate HE materials.

For rapid, facile, and low-temperature approaches to the synthesis of both bulk and nanoscale HE materials, atom-up strategies, where the HE material is constructed atom-by-atom, are therefore required.

## Atom-Up Synthetic Strategies for HE Materials

As discussed above, maximizing the configurational entropy of HE materials requires that the constituent elements are randomly and homogeneously distributed at the atomic level throughout the crystal lattice, which is difficult to achieve using traditional solid state routes. Several alternative approaches have therefore been reported for the synthesis of HE materials, including solvothermal synthesis, carbothermal shock synthesis, sol–gel synthesis, and molecular-precursor approaches. These atom-up approaches all share the use of atoms or molecules to atomically construct the high-entropy lattice, instead of altering prefabricated lattices.

**Figure 5:**
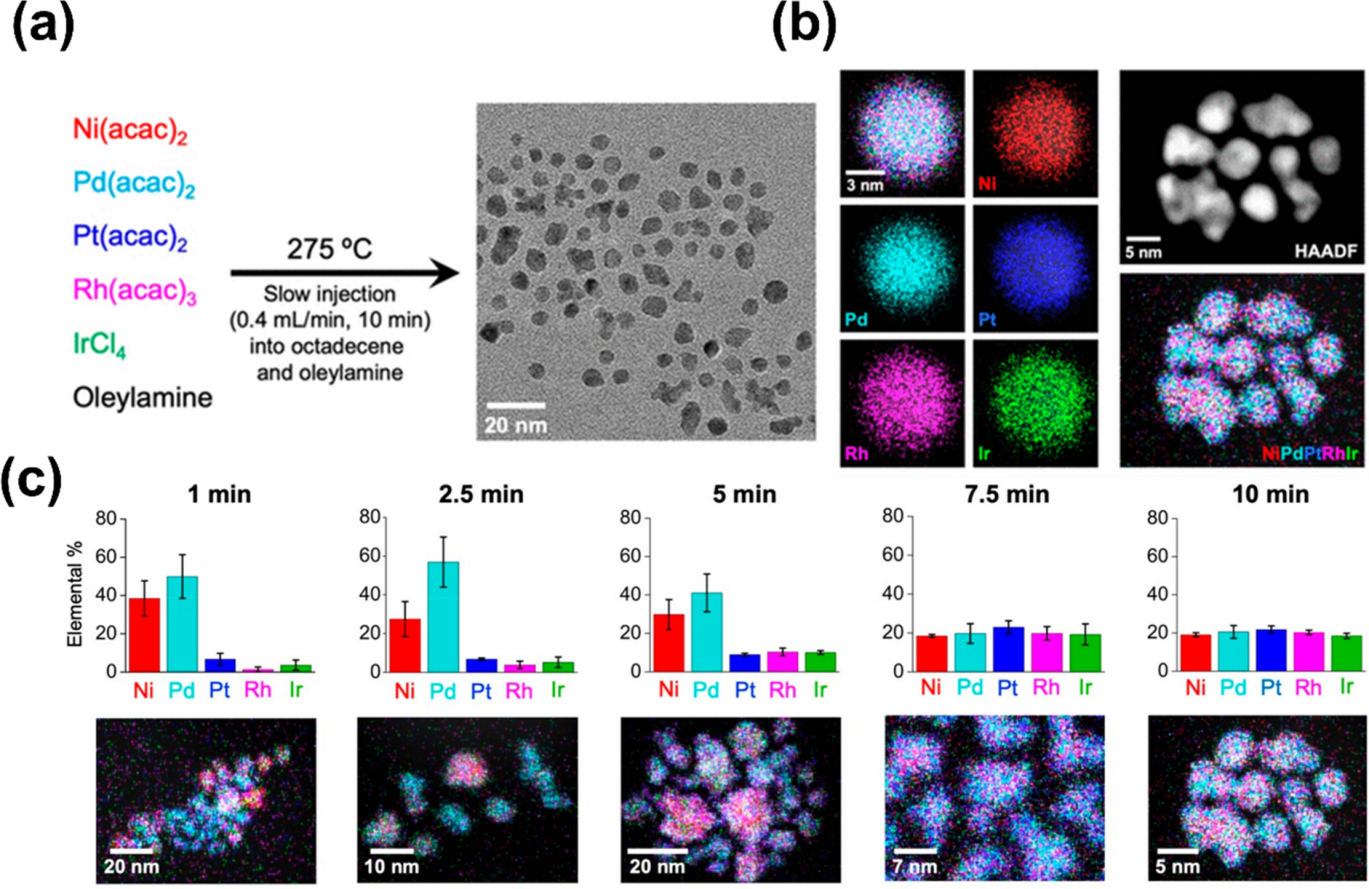
(a) Protocol for the synthesis of NiPdPtRhIr nanoparticles and the corresponding TEM image showing their morphology and size. (b) Microscopy characterization of NiPdPtRhIr nanoparticles. The STEM-EDS element map was overlaid along with individual STEM-EDS element maps (Ni Kα, red; Pd Lα, cyan; Pt Lα, blue; Rh Lα, pink; Ir Lα, green) for a single Ni_0.19_Pd_0.21_Pt_0.22_Rh_0.20_Ir_0.18_ nanoparticle. High-angle annular dark-field (HAADF)-STEM image and the corresponding overlaid STEM-EDX element map for an ensemble of Ni_0.19_Pd_0.21_Pt_0.22_Rh_0.20_Ir_0.18_ nanoparticles. Time-dependent formation of NiPdPtRhIr nanoparticles. Bar charts showing elemental composition (obtained from EDX measurements) and corresponding STEM-EDX element maps for samples of NiPdPtRhIr nanoparticles isolated at various times during the slow-injection synthesis (Ni Kα, red; Pd Lα, cyan; Pt Lα, blue; Rh Lα, pink; and Ir Lα, green). Adapted from ref (67).

Solvothermal synthesis is a chemical synthetic method for forming nanoparticles by undertaking a thermolytic reaction in a solvent.^65^ This method usually involves a high-boiling-point solvent that also acts as a capping agent for the synthesis of colloidal nanoparticles such as oleylamine/oleic acid or trioctylphosphine/trioctylphosphine oxide (TOP/TOPO). FeCoNiPtRu alloy nanoparticles with a size distribution of 3.5 ± 1.3 nm have been prepared in an oleylamine/oleic acid solution by a directed solvothermal synthesis, followed by a 290 °C solvothermal heat treatment for 8 min and rapid cooling (Figure 5).^66^ This technique has also been used to investigate, at a more fundamental level, the evolution of colloidal NiPdPtRhIr HE nanoparticles over the minute or few-minutes time scale during an oleylamine/octadecene solvothermal synthesis.^67^ This analysis showed that after 1 min Pd-rich PdNi seeds were formed, with minimal integration of the other elements, which is important for the autocatalytic addition of further elements.^68^ After 2.5 min, the Pd concentration in these seeds increased, with a slight decrease in Ni concentration and again minimal integration of the other elements. Only after 5 min was the concentration of Pt, Rh, and Ir seen to increase, with a concomitant decrease in the concentration of Pd, and a leveling out to near equimolar ratios of the elements after 7.5 min and no further change by 10 min (Figure 5c). Synthesis of other HE nanoparticles containing noble metals, transition metals and p-block metals was also undertaken, and it was shown that most systems formed near equimolar concentrations of elements within 10 min.^67^

This analysis demonstrates that HE nanoparticles are formed over very short time scales (ca. 7.5–10 min) compared to more traditional techniques such as elemental annealing (up to 110 h^61^). Fundamental analysis such as this is vital for establishing predictive pathways to achieve the rapid synthesis of phase-pure colloidal HE nanomaterials. While this synthetic strategy is similar to the core@shell synthetic route described above, the key difference is the formation of HE materials from the outset, as opposed to fabrication of low-to-medium entropy core@shell structures, with no true high-entropy component, followed by an annealing step to merge the core and shell structures into a single, homogeneous HE material.

Nebulized spray pyrolysis (NSP) also uses a solution of homogeneously mixed metal salts. In this method the solution is nebulized and carried to a hot wall reactor held at 1150 °C by flowing oxygen. In this method, the carrier gas is also the source of oxygen to fabricate a HE metal oxide lattice. The high entropy (CoCuMgNiZn)O and medium entropy (CoMgNiZn)O materials have been synthesized as rock salt structured nanoparticles of ca. 10 nm in size.^69^

Carbothermal shock is another atom-up approach that uses flash heating and cooling at temperatures of ∼2000 °C, over extremely short time scales of ∼55 ms and rapid ramp rates on the order of 10^5^ °C s^–1^. A solution of metal salts is deposited onto oxygenated carbon supports such as carbon fibers^70^ followed by rapid thermal treatment at high temperature.^71,72^ This method has been shown to be extremely versatile for the synthesis of nanoparticles with a range of unary, binary, ternary, quinary, hexernary, septernary, and octarnary compositions, the latter four of which can be classified as high entropy according to our definition.^70^

**Figure 6:**
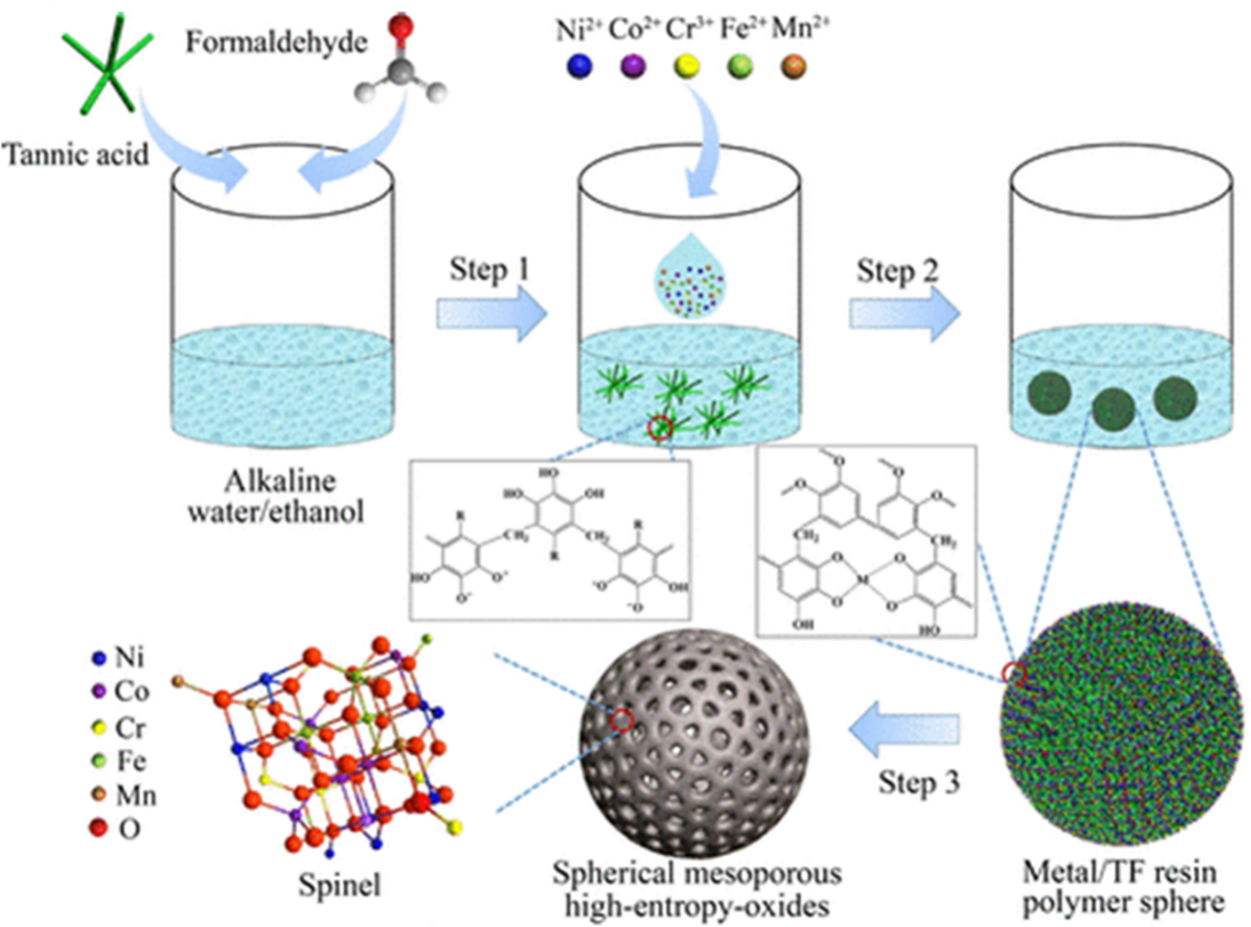
Schematic illustration of the sol–gel synthesis of mesoporous high-entropy oxide materials. Step 1: Formation of tannic acid/formaldehyde (TF) resin oligomers in the water/ethanol solvent using ammonia as a catalyst. Step 2: Formation of metal/TF resin polymer spheres. Step 3: Formation of spherical mesoporous HEOs by calcination of the polymer spheres in air. Adapted from ref (79).

Sol–gel synthesis is another interesting atom-up approach toward the synthesis of a multitude of size- and shape-controlled nanomaterials. The sol–gel process can roughly be defined as the conversion of a precursor solution into an inorganic solid by chemical means,^73−75^ and has been used as a synthetic approach to obtaining both high-entropy alloys^76^ and oxides.^77−79^ The synthesis of HE alloys can be conducted by initial dissolution of the metal salts, followed by addition of citric acid to form precursor complexes *in situ*. The solvent is then evaporated at 90 °C for 48 h to form a homogeneously dispersed gel, and the dried gels calcinated at 300 °C under an argon or flowing hydrogen atmosphere to obtain the alloys.^76^ The preparation of HE oxides can be carried out following a similar synthetic procedure, with the main difference that the metal salts are added to a solution containing Pluronic F127, tannic acid, and formaldehyde, neutralized with ammonia. After a 12 h solution homogenization and a 12 h solvothermal reaction at 100 °C, the resultant material was finally calcinated in air at either 400, 600, or 900 °C (Figure 6).^79^ Solvothermal, spray pyrolysis, carbothermal shock, and sol–gel synthetic methods are all examples of atom-up approaches as they exploit a homogeneously mixed solution of precursor ions prior to the construction of HE lattice, as opposed to more traditional techniques which require the deconstruction of multiple low-entropy crystal lattices prior to the fabrication of a single HE crystal lattice.

In our research group we favor an atom-up approach to building metal-sulfide crystal lattices using molecular metal xanthate and dithiocarbamate single-source precursors. This approach is particularly diverse and can be extended well beyond the synthesis of simple binary metal sulfides.^80^ By decomposing different precursors in tandem, it is possible to dope binary species (e.g., Ga/In doping into CdS),^81^ as well as to synthesize ternary (e.g., various phases of CuSbS)^82^ and higher metal sulfides (e.g., CuZnSnS).^80^ It is also possible to synthesize metal oxides by decomposing the precursors in air,^83^ or metal selenides and tellurides by exploiting different ligands to prepare the precursors.

**Figure 7:**
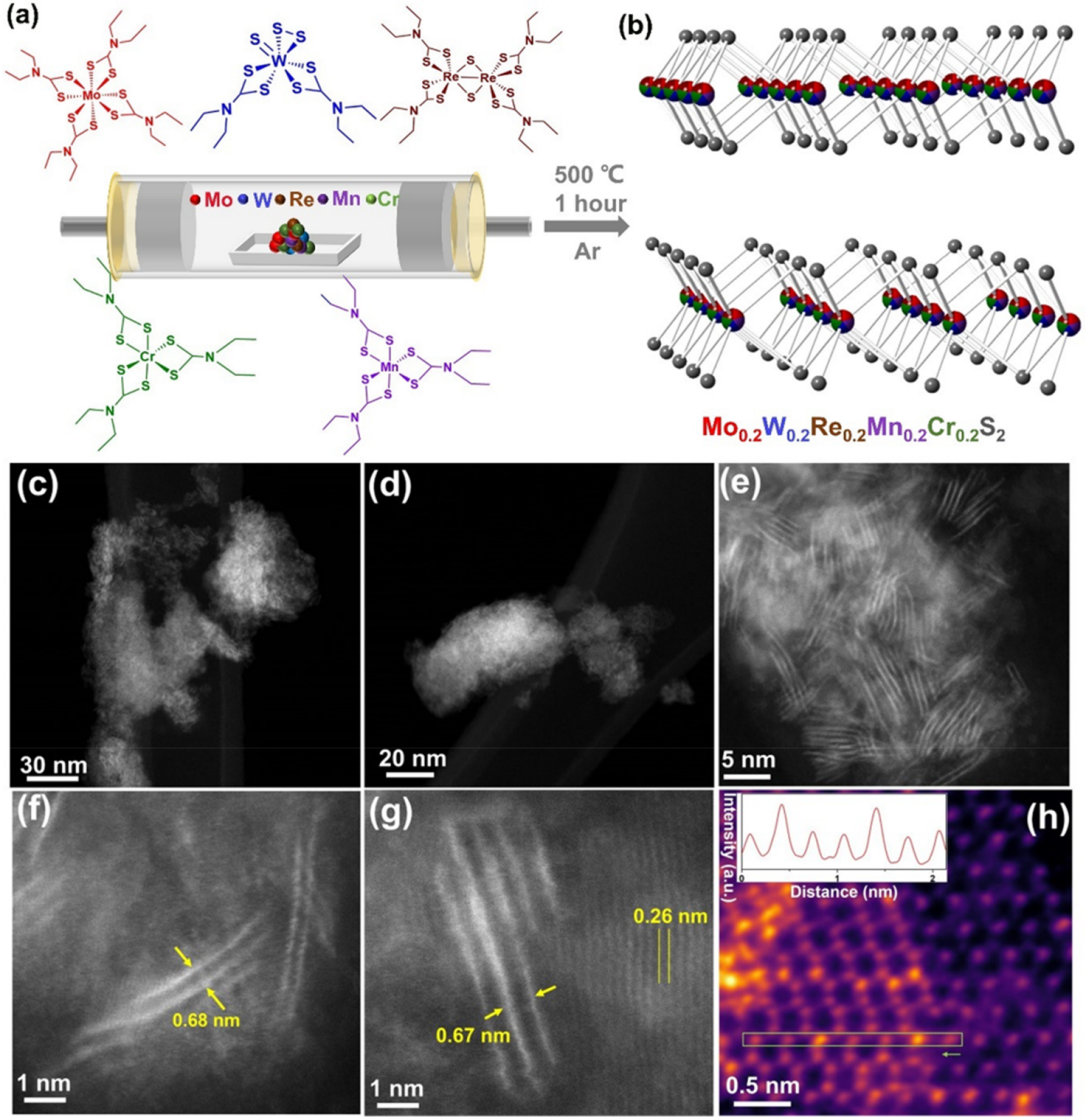
(a) Schematic illustration of the preparation of the high-entropy transition metal disulfide (CrMnMoWRe)S_2_, where five single-source precursors are decomposed in tandem to form the HE material. (b) Crystal structure of the HE transition metal disulfide showing variable occupancy of the metal sites within the 2H-MoS_2_ structure (indicated by the multicolored spheres). (c–h) STEM-HAADF images of exfoliated 2D nanosheets of (MoWReMnCr)S_2_ at various magnifications, showing various numbers of randomly distributed layers. Adapted with permission from ref (34) under a CC BY 4.0 license (https://creativecommons.org/licenses/by/4.0/).

The molecular precursor approach is ideally suited to the synthesis of high-entropy materials as it allows mixing, at the atomic level, of the precursors prior to synthesizing the HE lattice (Figure 7). By dissolving a combination of precursors in an appropriate solvent and evaporating the solvent, a homogeneous mixture of precursors is formed and subsequently decomposed in tandem, building the HE lattice atom-by-atom. The molecular precursor approach also has a major advantage in that the M–X bonds (where M is the metal and X the chalcogen) are prefabricated prior to decomposition of the precursor and formation of the metal–chalcogenide lattice, thus avoiding side reactions.^84^ The molecular precursors are typically nontoxic and air stable, and the rates and temperatures of decomposition are tunable by altering the chemistry of the precursors, making this a scalable and versatile technique.^85,86^ The decomposition of the precursors is usually rapid and occurs at a low temperature, typically requiring heating between 300 and 500 °C for 1 h. Furthermore, the library of molecular precursors available from the transition, main, and even lanthanide groups means that high-entropy materials can potentially be formed from a broad range of elements.^84^

**Figure 8:**
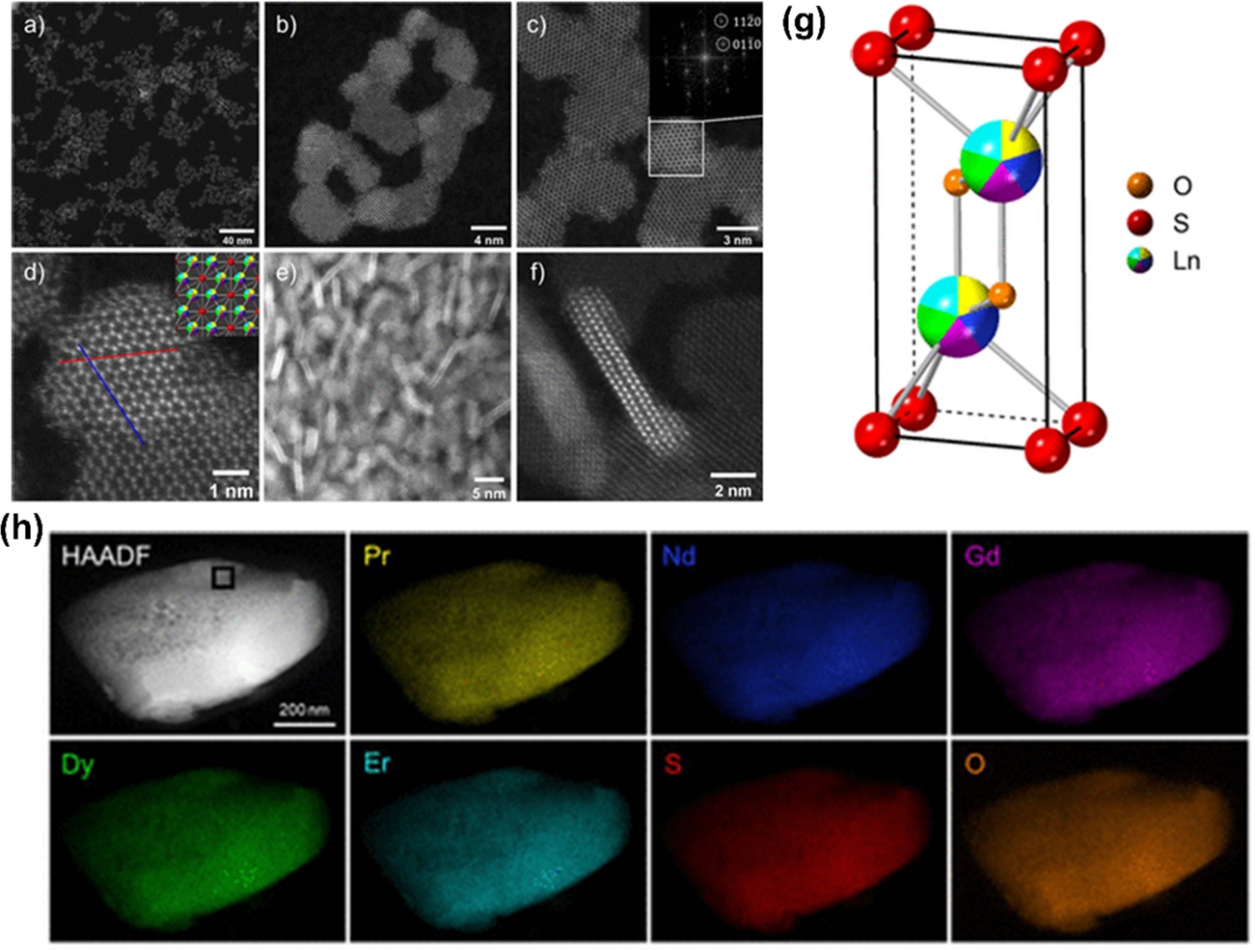
HAADF-STEM images of HE Ln_2_SO_2_ nanoparticles at lower (a, e) and higher magnifications (b–d, f). The inset in (c) shows the FFT image of the highlighted area, demonstrating that the particle is being viewed along the [0001] direction, and the inset in (d) shows the crystal structure viewed along the same direction as the STEM image. Adapted from ref (88). (g) Predicted crystal structure of the HE Ln_2_SO_2_ material, where “Ln” represents the high-entropy site and has split occupancy between Pr, Nd, Gd, Dy, and Er in equimolar ratios. (h) STEM EDX intensity maps of a single particle of the 5 lanthanide sample at different length scales. Each element is relatively evenly smeared across the particle and where variation is observed, and it is systematic across each element map, suggesting it is caused by variations in particle thickness in those areas. Reprinted from ref (87).

We initially proved this concept for HE materials by synthesizing HE lanthanide oxysulfides (Ln_2_SO_2_).^87^ Through the precursor approach, the synthesis of HE lanthanide oxysulfide lattices requires temperatures of 900 °C and annealing times of 5 h to obtain crystalline materials. In this case, the relatively high temperature and long synthetic time is due to the unique chemistry of the lanthanide oxysulfides.^87^ Contrarily, the use of transition-metal dithiocarbamate precursors allows for significantly lower temperatures and shorter annealing times (500 °C, 1 h).^34^ The use of xanthate (S_2_COR), rather than dithiocarbamate (S_2_CNR_2_), based precursors would significantly lower the synthetic temperature further to between 200 and 350 °C.^86^ Following this proof-of-concept, we further expanded the precursor approach to the synthesis of nanoscale HE materials by using a solvothermal approach to obtain HE lanthanide oxysulfide nanoparticles, which were found to show quantum confinement behavior when compared to their bulk counterparts.^88^ We also used this method to synthesize a 2D HE MoS_2_-structured (CrMnMoWRe)S_2_ transition-metal dichalcogenide, which was found to be significantly more catalytically active than the parent MoS_2_ due to the presence of first-row transition metals within the 2H MoS_2_ lattice structure (Figure 8).^34^

## Summary and Outlook

High-entropy materials are already of significant interest for a wide range of applications, including energy conversion and storage, catalysis, and thermoelectrics, and we expect these materials to become even more important in the future as they are diversified away from alloys and toward multilattice structures such as chalcogenides, MXenes, perovskites, and spinels. It can be demonstrated with simple mathematical models that the chemical space in which high-entropy materials exist is so huge that it could potentially dwarf the number of all of the known compounds that have been discovered to date, and there will no doubt be new families of high-performance HE materials discovered over the next few years with new applications following.

Since the inception of inorganic HE materials, a multitude of synthetic approaches have been reported and the possibilities for future progress in this area are exciting. In this Perspective, we have compared top-down vs bottom-up synthetic strategies, with a view to highlighting the advantages of atom-up over top-down methods. Although inevitably nonexhaustive, we have chosen a range of examples to illustrate the range synthesis methods used at present.

It is our view that, in order to fully realize the advantages of HE materials conferred by their high configurational entropy, homogeneous mixing of the elements at the atomic scale is vital and, by definition, should be the benchmark by which synthetic methods are judged. Why this is so vital in a statistical sense can be understood considering the number of unique atomic sites within a high-entropy material: if the atoms are not perfectly mixed, then there are two major downfalls: (i) the system will not be maximally disordered and will therefore not fully benefit from entropic stabilization; and (ii) the number of theoretical unique sites drops exponentially, which can affect performance in certain applications, in particular, catalysis.

With traditional solid-state routes, true mixing at the atomic scale is often difficult to achieve without extremely long reaction times. This is partly due simply to the size of the starting material powders, which generally have particle sizes in excess of 100 nm, even with the best milling, but also to the inevitable variation in the diffusion coefficients of individual metals or anions within the lattice during the solid-state reaction and to the thermodynamics dictated by the high-temperature conditions and long reaction times. It is likely that the potential-energy landscape during synthesis of HE materials has a large number of local minima corresponding to all of the single-component, binary, ternary, quaternary, etc. compositions that can potentially form as well as the HE lattice. We therefore believe that chemical routes utilizing molecules to build the lattices atom-by-atom with predictable reactivity are optimal to produce true high-entropy materials with maximum disorder, which gains both kinetic (avoiding prefabricated lattices with local energy minima) and thermodynamic (*S*_conf_) favorability. From this perspective, sol–gel routes to high-entropy oxides, and the use of molecular precursors to form chalcogenides and pnictides in particular, are both extremely promising routes that need to be further developed, as both start from molecular species that can be mixed at the atomic scale by solubilization prior to reaction.

However, we note that in much of the current literature, and in particular for inorganic HE materials, there appears to be an overall relatively poor understanding of the atomic-scale mixing and how to optimize this to produce entropy-stabilized compounds. For example, in many cases materials are characterized with EDX spectroscopic maps that only show elemental distributions at the microscale, and this does not provide unambiguous evidence of homogeneous mixing at the atomic scale. From the discussion in this Perspective one can see why this is problematic, yet these issues persist—techniques at or close to lattice resolution, such as STEM-EDX imaging, are needed to unambiguously assess the elemental distributions, and any study that does not include such characterization should, in our view, be treated with skepticism (*nullius in verba*). However, we do acknowledge that there are some excellent examples and guidance of atomic scale characterization of HE and multicomponent materials,^89−91^ in particular with the use of atom probe tomography.^92,93^ These examples can be used as models for good practice in characterizing HE materials in future.

Beyond synthesis, we currently see two further challenges to this field. The first is a consequence of the chemical complexity of these materials. We have demonstrated to the reader that these materials are of significant complexity, with the possibility of unique sites within a high-entropy material almost unimaginable (we estimated 10^33^ unique sites in a 6-component material when third-nearest neighbors contribute to a unique site, *vide supra*). Contemporary materials science benefits heavily from computational modeling to predict and understand physical properties, but approaches for modeling disordered systems are comparatively less well developed than those for ordered structures.

Current methodology can be broadly divided into four classes, *viz*.: (1) linearly combining the properties of suitable endmember structures, as in the virtual-crystal approximation (VCA);^94^ (2) systematically enumerating configurations with a given composition in a chosen supercell of a common parent structure, as implemented for example in the popular Site-Occupation Disorder (SOD) code;^95^ (3) generating a single structure or set of disordered structures that best represent a fully disordered material using a technique such as the special quasi-random structure (SQS) approach;^96^ and (4) using explicit calculations on a subset of configurations to parametrize a model Hamiltonian to calculate the energetics of a more complete set, as in the cluster-expansion method.^97,98^

In the context of HE materials, each of these may have its own advantages and disadvantages. Methods such as the VCA are low cost and may be appropriate for modeling bulk properties such as transport coefficients that are measured over larger length scales. Approaches of this kind have, for example, been successfully applied to study the electronic structure of Sn/Ge alloys^99^ and the lattice thermal conductivity in Sn(S,Se) alloys.^100^ Techniques based on representative models allow the properties of individual atomic sites to be explicitly investigated, which is important for modeling catalytic activity, as in, for example, our recent work on the hexernary (MoWReMnCr)S_2_ system.^34^ However, given the complexity of HE materials, it is doubtful whether a single model of a size amenable to electronic-structure calculations can, in general, capture a fully representative set of sites. Methods that consider a large number of configurations provide access to thermodynamic properties such as the enthalpy, entropy, and free energy of mixing, and have, for example, successfully been applied to the binary Sn_*x*_(S,Se)_*y*_ systems.^101,102^ However, given the number of unique sites in a high-entropy material, it is easy to imagine that choosing an appropriate supercell, enumerating the unique configurations, and/or parametrizing a model Hamiltonian for a cluster-expansion model would be far more challenging than for these binary solid solutions.

Given the potential for modeling to aid our understanding of the unique properties of HE materials, we propose the development of suitable modeling approaches as an important complementary research direction to synthesis and characterization protocols. One possible avenue is the exploitation of artificial intelligence (AI) for scaling atomistic simulations to more realistic supercells, building on recent successes in developing machine-learned force fields.^103,104^

It is worth noting here that the issue of how to fully characterize such complex materials in a way that is representative of the whole sample is also an issue for experiments. Due to the unique number of sites, we would argue that multiscale characterization, combining lattice-resolution techniques with nanoscale, microscale, and mm/cm-scale microscopies, as appropriate, and bulk techniques such as transport measurement, is *de rigueur* when working with these materials.

The second grand challenge we foresee is the development of equilibrium phase diagrams for these materials. This again is challenging, due to the multidimensional nature of the chemical space, but would be extremely informative for development of optimized synthetic routes and would provide a framework for establishing the thermodynamics of when and how a high-entropy system becomes entropically stabilized; this is currently poorly understood, aside from a small number of studies on specific chemical systems.
